# Does Breast Implant Size Larger Than Mastectomy Specimen Size Increase the Risk of Flap Necrosis?

**DOI:** 10.1093/asjof/ojag063

**Published:** 2026-05-15

**Authors:** Lisa B Cassileth, Kelly Killeen, Amy York, Dorian Rosen

## Abstract

**Background:**

Mastectomy skin flap necrosis (MSFN) is a critical postoperation complication in postmastectomy breast reconstruction. High replacement tissue expander fill volume and large mastectomy specimen size are commonly assumed to increase MSFN risk. Thus, a common recommendation in the practicing surgeon's office is not to increase breast volume during reconstruction. However, there is minimal literature on the question of whether postsurgical large or larger breast size is a risk factor for MSFN.

**Objectives:**

This study asked whether risks of MSFN and other complications were increased when an implant larger than the size of the mastectomy specimen was placed during direct-to-implant (DTI) reconstruction for nipple-sparing mastectomy (NSM).

**Methods:**

A 9-year retrospective review of immediate DTI breast reconstruction by 2 surgeons at a private clinic was conducted encompassing a total of 499 breasts in 270 consecutive adult female patients. Compared were patients with breasts that had implants placed greater in size in cubic centimeters than the weight of mastectomy specimen in grams (the upsize group, n = 361) vs those that had smaller implants placed (the downsize group, n = 138).

**Results:**

MSFN was nonsignificantly lower in the upsize group compared to the downsize group (3.59% vs 4.35%, *P* = .6966). When secondary outcomes differed significantly, complications were higher in the downsize group. BMI, but not size difference between specimen and implant, significantly predicted MSFN in logistic regression modeling.

**Conclusions:**

Increasing breast size was not associated with increased risk of MSFN after NSM with DTI reconstruction.

**Level of Evidence:**

3 (Therapeutic) 
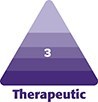

Breast reconstruction after prophylactic or therapeutic mastectomy is increasingly common: A recent retrospective analysis of 197,387 individuals with breast cancer in the National Surgical Quality Improvement Program database found that the desire for postmastectomy reconstruction using implants doubled over 12 years.^1^ Implant-based reconstruction is used in ∼80% of reconstructions performed after mastectomy.^2^ Immediate breast reconstruction, in which the reconstruction is performed during the same surgery as the mastectomy, may be accomplished by a 2-stage tissue expander (TE) reconstruction, or a single stage direct-to-implant (DTI) approach. TE reconstruction is more commonly performed and boasts a larger body of research than DTI.^3,4^ Nonetheless, DTI has been widely used for more than 2 decades and has been well studied by us and others.^5-7^. New technologies, including acellular dermal matrix (ADM) and real-time perfusion mapping, have made DTI safer and more accessible.^8,9^ DTI is especially well suited to prophylactic surgery due to aesthetic, functional, and logistical patient needs.^10,11^ Some comparative analyses have suggested that DTI is associated with higher rates of certain complications, including mastectomy skin flap necrosis (MSFN).^2,12^ Others, including a recent prospective cohort study, did not find increased risk of complications in reconstructions performed with DTI compared to TE.^4,13^

DTI carries several advantages over TE, such as the single surgical exposure, fast recovery, less patient discomfort including less impact on upper extremity function, and better aesthetic results.^10,14,15^ One disadvantage of DTI is that there are relatively narrow criteria for both candidates and reconstruction options.^15^ According to current recommendations, the ideal DTI recipient is a relatively young, thin nonsmoker with small, nonptotic breasts, who does not desire a larger postreconstruction breast volume.^15^ Although there is little direct evidence against it, increasing breast size is a concern in DTI, due to tissue elastic capacity and because larger initial expander volume increases MSFN risk in analogous studies of TE reconstruction.^16-18^ Risk factors for post-DTI complications include age, high BMI, radiotherapy exposure, history of cardiovascular disease, ptosis, large breast volume, and smoking.^19,20^ Since many of these factors are nonmodifiable, there is great potential to improve patient safety by interrogating the reconstructive approach in detail. Furthermore, current decision-making frameworks may unnecessarily exclude patients who would benefit from the advantages of DTI and are not truly at elevated risk of complications. More study is needed to identify modifiable drivers of poor outcomes after DTI reconstruction.

As the breast cancer treatment paradigm has shifted from a “maximum tolerable” to a “minimum effective” approach, conservative mastectomy approaches, such as skin-sparing mastectomy (SSM) and nipple-sparing mastectomy (NSM), are now standard.^21^ Technical advancements have enabled NSM to attain cure rates equivalent to traditional SSM.^2^ Beyond its efficacy, NSM may have unique benefits for psychosocial and sexual health.^22-24^ There is some evidence that NSM carries a higher risk of complication than SSM after implant-based breast reconstruction, whether TE or DTI.^2,25^

MSFN is a major concern after DTI, since it can cause implant loss, aesthetic degradation, and delays in adjuvant therapy.^26^ The pathophysiology of MSFN is incompletely described, but 1 contributor is oxidative damage downstream of dysfunctional microvascular flow and ischemia–reperfusion injury.^27^ In both clinical studies of MSFN and experimental animal models of skin flap necrosis, intraoperative microcirculatory stasis correlates closely with necrotic lesion size.^28-30^ A recent prospective analysis of 53 breast reconstructions found that intraoperative mastectomy skin flap hypoperfusion, determined by indocyanine green (ICG) imaging, has strong positive and negative predictive value for development of MSFN within 3 months.^31^ For the DTI reconstructions evaluated in that study, perfusion assessment was performed after a sizer was inserted and the skin temporarily closed, meaning that authors could assess the effect of implant size on skin flap perfusion. Such methodology allows assessment of the interaction between implant volume and perfusion, since implant volume may affect the microvascular flow, changing the flap necrosis rate. In daily practice, mastectomy flaps are often overly thin, overdissected, and sustain retraction injury during the mastectomy itself. This places the plastic surgeon's decision regarding implant size of utmost importance, as excessive increases in breast volume, whether by TE or DTI reconstruction, may place patients at increased risk of MSFN by compressing skin flap vasculature, interrupting oxygenation, and compromising tissue integrity. This study elucidates surgical factors influencing MSFN risk.

In our practice, many patients wish to increase their breast size during postmastectomy reconstruction. We hypothesized that study participants who received a larger implant size than mastectomy specimen size would have higher rates of MSFN. The primary comparison of interest was downsize vs upsize group. The primary outcome of interest was MSFN requiring surgery. Our research aimed to illuminate important understudied questions in postmastectomy breast reconstruction: What are the most impactful reconstruction-related factors to consider when calculating complication risk? What is the range of possibility for safe implant-based reconstruction? Ultimately, how do we tailor those risk calculations to individuals?

## METHODS

### Study Design

This single-center, 5-year retrospective cohort study enrolled consecutive patients undergoing therapeutic or prophylactic mastectomy with immediate DTI reconstruction over 9 years at a private clinic. Inclusion criteria selected for adult females undergoing unilateral or bilateral mastectomy with DTI reconstruction. Patients with prior radiation, prior augmentation, necrosis from methylene blue injection, or those desiring concomitant mastopexy were excluded. Data were de-identified prior to use in this study. The study protocol was approved by the Western Institutional Review Board-Copernicus Group Institutional Review Board (reference number: 20252331).

### Sizing Determination

The decision to upsize or downsize was patient directed. To inform decisions on desired breast volume, patients underwent 3-dimensional imaging with the Vectra system (Canfield Scientific). Patients desiring to upsize also tried on sizers to assess the desired volume.

### Surgical Approach

All patients underwent inferior mammary crease approaches. Mastectomy was performed by a breast surgeon, and reconstruction was performed by a plastic surgeon. Incision was marked by the plastic surgeon prior to surgery. Local anesthesia (lidocaine with epinephrine) was injected around the incision prior to incision. After incision with a #15 blade, the mastectomy was performed using Bovie cautery (Apyx Medical, Clearwater, FL), with the exception of sharp dissection directly under the nipple and areola complex. Tumescent technique was not used. Mastectomy specimens were weighed.

After completion of mastectomy, hemostasis was achieved using Bovie cautery. The pectoral muscle was not dissected. ADM or poly-4-hydroxybutyrate mesh (P4HB) was utilized for implant support. ADM was sutured to the pectoral fascia using 2-0 PDS (Ethicon Inc., Raritan, NJ), with the implant placed using a Keller funnel (Allergan Aesthetics, Irvine, CA) between the pectoral muscle and dermal matrix. For cases using P4HB, the implant was placed into a mesh construct before placing it into the patient, suturing it closed with 2-0 Vicryl (Ethicon Inc., Raritan, NJ). The implant with the mesh was then placed in the prepectoral space. 2-0 Vicryl was used deep to the inframammary crease to secure the mesh to the chest wall. Sizers were used based on patient/surgeon preoperative discussion. With the sizer in place, the patient was seated upright. Incision tension was checked, and all incisions were deemed low tension before final size was chosen. Patients were upsized or downsized depending on the preoperative plan. Mastectomy edge incision was trimmed to remove traction injury skin and check for perfusion. A Prevena VAC (3M Health Care, San Antonio, TX) designed for closed incisions was utilized on all cases. A single drain was used for each mastectomy. Drains were removed at 30 cc/day or at 2 weeks. The majority of patients had postoperative visits at 1 day, 1 week, 1 month, and 6 months. Some patients were followed at initial intervals of weekly or greater depending on complication rates.

### Analytic and Statistical Approach

Mastectomy weight was used to approximate volume in order to assign patients to upsize vs downsize groups. Breast tissue density is variable, but an average density of ∼1.06 has been reported, making breast weight a good approximation of breast volume.^32^ To control for the potential confounding contribution of BMI, matched-pairs analysis was performed. BMI was matched to within 1 kg/m^2^. To assess the effect of large implant size, the population was separated into those who received implants larger than 500 cm^3^ vs those who received implants smaller than 500 cm^3^.

Analysis was performed on a per-breast basis. An alpha value of *P* < .05 was set as the cutoff for statistical significance. Normality was assessed using Anderson–Darling normality test. For comparison of means, *t* test or Mann–Whitney *U* test was used depending on whether data were normally distributed. Correlation was assessed by Spearman's rank correlation. Categorical comparisons were made using chi-square test. Logistic regression was used to model multivariable effects. Statistical analyses were performed using R statistical software (ver. 4.3.2), GraphPad Prism (ver. 10.4.0), and G*Power (ver. 3.1.9.6). Data visualization was performed in GraphPad Prism.

## RESULTS

This study analyzed a total of 499 breasts from 270 individuals. The cases examined in this study were performed between January 19, 2015, and November 25, 2024. Of the 499 breasts included in the study, 193 breasts (38.7%) were removed for invasive breast cancer or ductal carcinoma in situ (DCIS). The remaining breasts (61.3%) were removed for prophylaxis, either in a bilateral reconstruction when unilateral cancer was present or for high-risk patients with no evidence of cancer. Two patients had bilateral cancer. The population was relatively young and healthy, with an average age of 44.8 years (range: 19-74 years), and no major illness aside from breast cancer diagnosis. Directionality of size change was decided preoperatively, and it was not necessary to change a patient that had desired an upsize to a downsize because of mastectomy flap perfusion. In some cases, patient's goal size was not achieved. In the upsize group, observed flap tension sometimes limited increase. In the downsize group, patient's skin redundancy limited the degree of desired downsizing. Silicone implants were used in 99.6% of cases.

**Figure 1.**
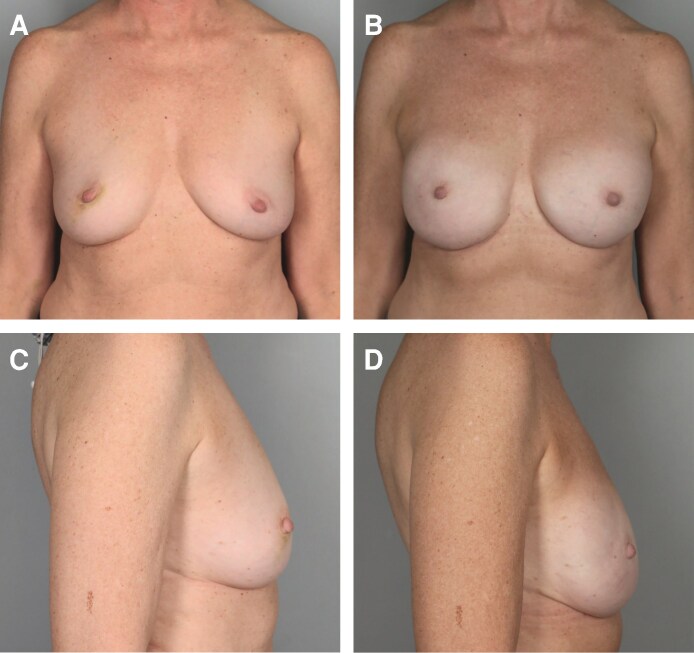
Illustration of upsizing. A 57-year-old female with right ductal carcinoma in situ (DCIS). (A) Preoperative frontal view and (B) 8 months postoperatively following nipple-sparing mastectomy (NSM) (mastectomy specimen 187 g right, 153 g left; implants 360 cm^3^ bilateral) frontal view. (C) Preoperative profile view and (D) 8-month postoperative profile view.

**Figure 2.**
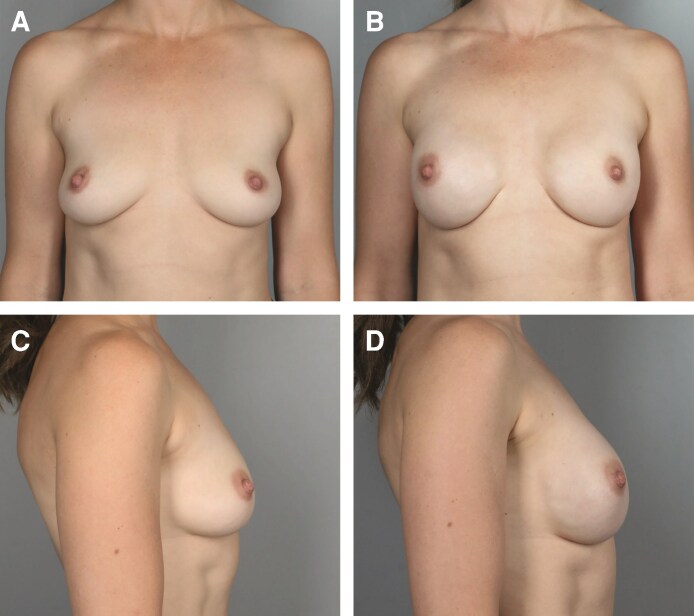
Illustration of upsizing. A 34-year-old female with breast cancer type 1 susceptibility protein (BRCA1) mutation. (A) Preoperative frontal view and (B) 11 months postoperatively following nipple-sparing mastectomy (NSM) (mastectomy specimen 133 g right, 149 g left; implants 245 cm^3^ bilateral) frontal view. (C) Preoperative profile view and (D) 11-month postoperative profile view.

**Figure 3.**
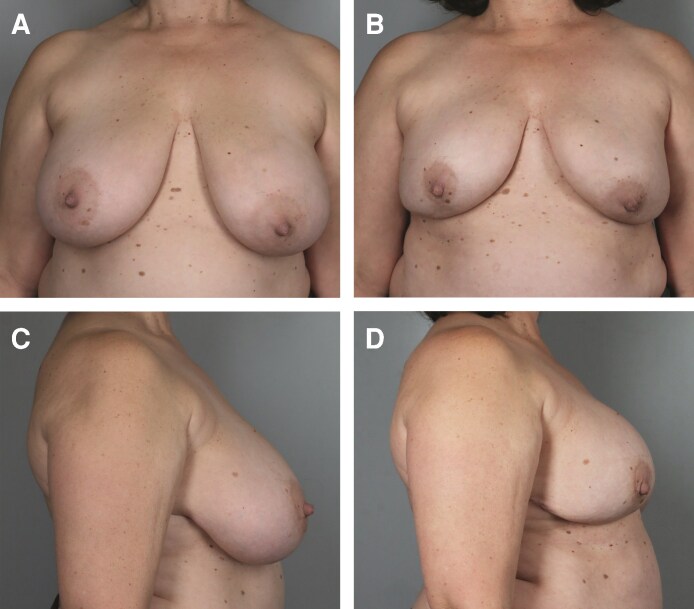
Illustration of downsizing. A 58-year-old female with BRCA1 mutation. (A) Preoperative frontal view and (B) 16 months postoperatively following nipple-sparing mastectomy (NSM) (mastectomy specimen 686 g right, 668 g left; implants 405 cm^3^ bilateral) frontal view. (C) Preoperative profile view and (D) 16-month postoperative profile view.

**Figure 4.**
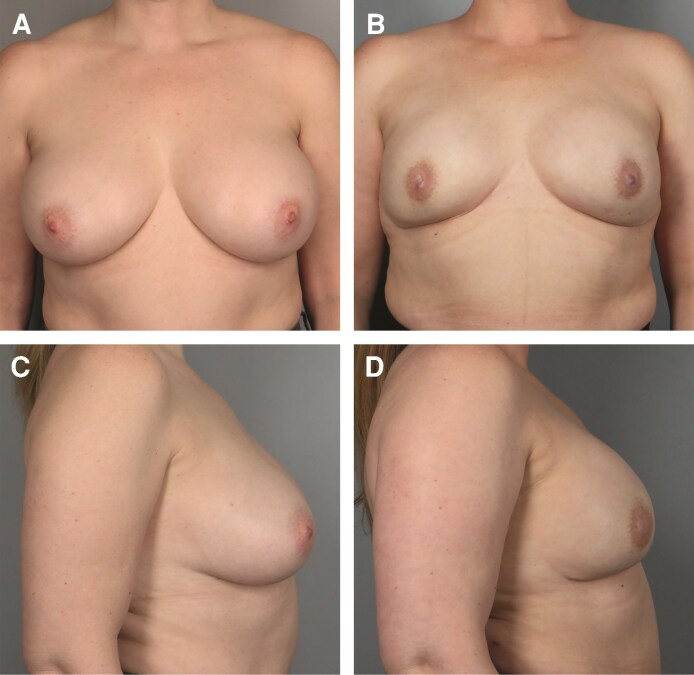
Illustration of downsizing. A 36-year-old female with right ductal carcinoma in situ (DCIS) and breast cancer type 2 susceptibility protein (BRCA2) mutation. (A) Preoperative frontal view and (B) 16 months postoperatively following nipple-sparing mastectomy (NSM) (mastectomy specimen 655 g right, 670 g left; implants 445 cm^3^ bilateral) frontal view. (C) Preoperative profile view and (D) 16-month postoperative profile view.

The median follow-up time was 12.40 months. A total of 361 breasts were upsized, with typical results shown in Figures 1, 2. A total of 138 breasts were downsized, with typical results shown in Figures 3, 4. The average implant size was 122% of specimen volume for the upsize group and 70% for the downsize group. The upsize and downsize groups were comparable in age, cancer incidence, chemotherapy, and radiation exposure, while BMI (22.9 vs 24.7, *P* < .0001) and specimen size (279 g vs 507 g, *P* < .0001) were significantly higher in the downsize group (Table 1). No study participants were current smokers.

**Table 1.** Population Characteristics

	Downsize	Downsize SD	Upsize	Upsize SD	*P* value
Number	138		361		
Age (years)	45.3	11.4	44.4	9.9	.262
BMI (kg/m^2^)	24.7	4.3	22.9	4.3	<.0001
Breast cancer %	39.9		38.2		.7384
Post-op chemo %	10.9		10		.7673
Radiation %	10.3		9.2		.7325
Specimen size (g)	507	228	279	137	<.0001
Implant size (cc)	411	151	384	136	.1011

Mann–Whitney *U* test was used to compare means. Chi-square test was used to compare the rates of binary outcomes.

**Figure 5.**
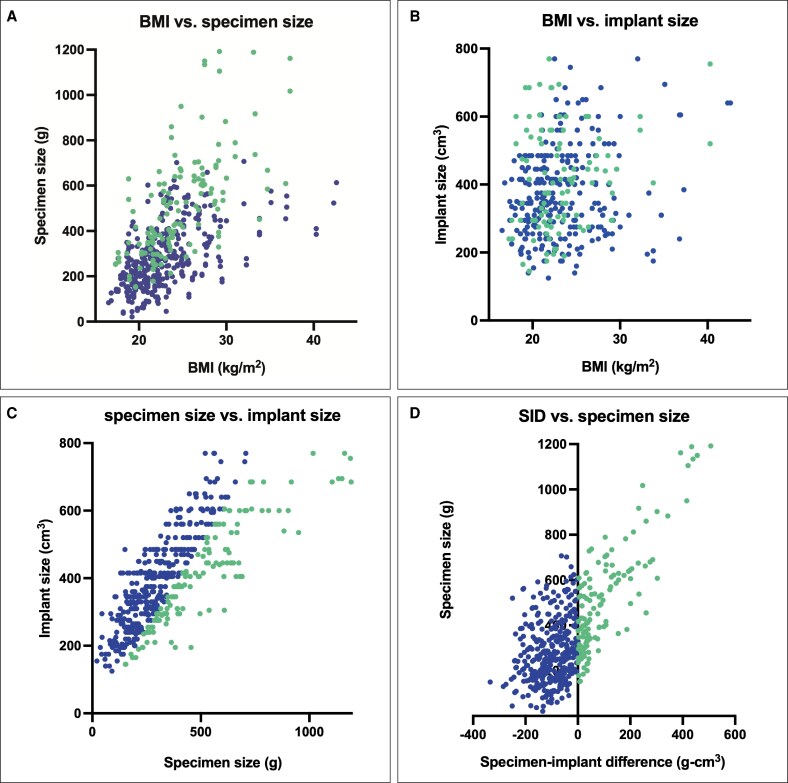
Correlations of BMI, specimen size, and implant size. (A) BMI vs specimen size. (B) BMI vs implant size. (C) Specimen size vs implant size. (D) SID vs specimen size. SID, specimen–implant difference, ie, specimen size in grams minus implant size in cubic centimeter.

BMI correlated significantly with specimen size (*P* < .0001), and the Spearman r_s_ correlation coefficient was 0.6162, indicating moderate correlation (Figure 5A).^33^ BMI correlated significantly but very weakly with implant size (*P* < .0001, r_s_ = 0.1764) (Figure 5B). Specimen and implant size correlated strongly with each other (*P* < .0001, r_s_ = 0.7924) (Figure 5C). Specimen size and specimen–implant difference (SID) correlated moderately (*P* < .0001, r_s_ = 0.5396) (Figure 5D).

**Figure 6.**
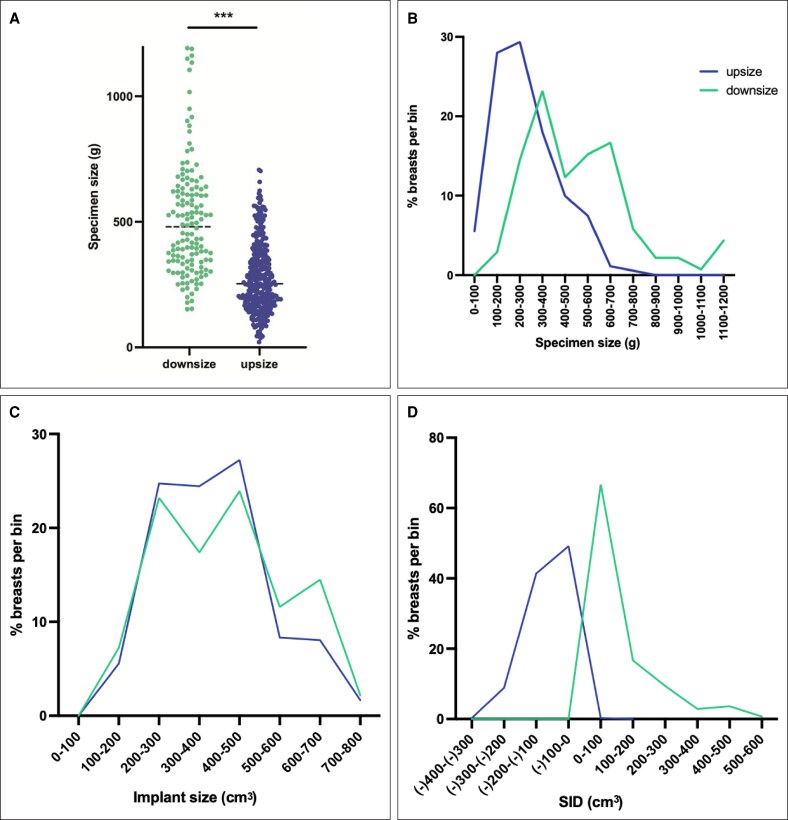
Distribution of specimen and implant size by group. (A) Individual specimen size values. ***P < .0001. (B) Histogram of specimen size by 100 g bin. (C) Histogram of implant size by 100 cm^3^ bin. (D) Histogram of specimen–implant difference.

When comparing specimen size in g to implant size in cm^3^, downsizing involved an average change of −96±112 cm^3^ (range: −1, −507), while upsizing involved an average change of 104±64 cm^3^ (range: 0, 335). Specimen size was significantly larger (279 g vs 507 g, *P* < .0001) in the downsize group (Figure 6A), and the distribution was notably wider in the downsize group than the upsize group (Figure 6B). The distribution of implant size was very similar between upsize and downsize groups (Figure 6C). Specimen-implant difference was primarily within ±200 in both groups, with 90.2% of upsizers and 83% of downsizers falling within this range (Figure 6D).

**Figure 7.**
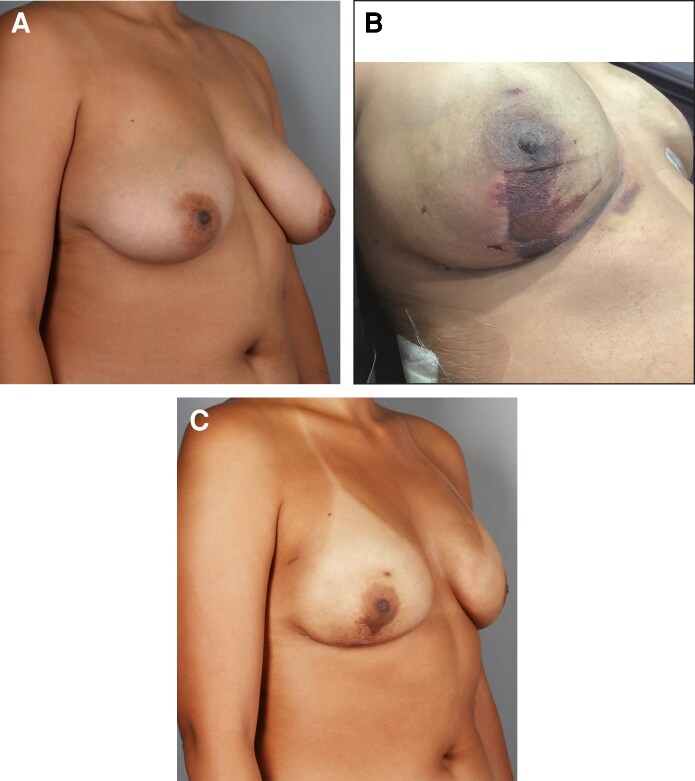
Illustration of mastectomy skin flap necrosis (MSFN). (A) Preoperative photograph of a 30-year-old female with left invasive ductal carcinoma, downsized (mastectomy specimen 622 g right, 680 g left; implants 485 cm^3^ bilateral). (B) Postoperative MSFN occurred 1 week postoperatively in this patient and was immediately excised and closed. (C) Four months postoperatively.

Chi-square test was used to interrogate how directionality of size change associates with outcomes (Table 2). Overall, the rate of outcomes hewed closely to the proportionality of the underlying sample. There was no effect of downsizing vs upsizing on the primary outcome of MSFN (4.35% in the downsize group vs 3.59% in the upsize group, *P* = .6966). The appearance of MSFN is illustrated in Figure 7. There was no effect of downsizing vs upsizing on the secondary outcomes seroma (13.8% vs 17.5%, *P* = .3206) or hematoma (2.9% vs 3.1%, *P* = .9231). Dehiscence was significantly elevated in the downsize group (4.4% vs 1.1%, *P* = .0209). Incidence of infection requiring surgery and implant loss was significantly higher in the downsize group (2.9% vs 0.6%, *P* = .0316), though the overall incidence of this complication was very low (1.2%).

**Table 2.** Chi-square Results: Downsize vs Upsize Groups, Primary and Secondary Outcomes

	Overall %	Downsize (n)	Upsize (n)	*P* value
Total		138	361	
MSFN	3.8	6	13	.6966
Seroma	16.4	19	63	.3206
Hematoma	3.0	4	11	.9231
Dehiscence	2.0	6	4	.0209
Infection w/o surgery	3.2	9	7	.0095
Infection w/surgery and implant loss	1.2	4	2	.0316

MSFN, mastectomy skin flap necrosis.

Because the downsize group had a higher average BMI overall, a matched-pairs analysis was performed to control for BMI. A total of 131 downsize–upsize pairs were matched by BMI, and this subset of the study population was re-analyzed by chi-square test (Table 3). Rates of MSFN were identical (3.9%), and there were no statistically significant differences in rates of other outcomes. The only statistically significant difference in the BMI-matched population was specimen size, which was 37% larger in the downsize group (317 g vs 501 g, *P* < .0001).

**Table 3.** Population Characteristics and Chi-square Results of BMI-Matched Subpopulation

	Downsize	Downsize SD	Upsize	Upsize SD	*P* value
BMI-matched population statistics	131		131		
Age (years)	45.1	11.6	43.3	9.8	.1255
BMI (kg/m^2^)	24.4	4.1	24.3	4.0	.9792
Breast cancer %	38.9		42.0		.6146
Post-op chemo %	10.9		10.0		.7673
Radiation %	10.0		12.2		.5694
Specimen size (g)	501	222	317	133	<.0001
Implant size (cc)	409	149	417	130	.445
Outcomes					
MSFN	5		5		>.9999
Seroma	28		17		.0716
Hematoma	4		3		.717
Dehiscence	2		3		.6516
Infection w/o surgery	3		3		.6516
Infection w/surgery and implant loss	5		6		.758

Mann–Whitney *U* test was used to compare means. Chi-square test was used to compare the rates of binary outcomes.

MSFN, mastectomy skin flap necrosis.

**Figure 8.**
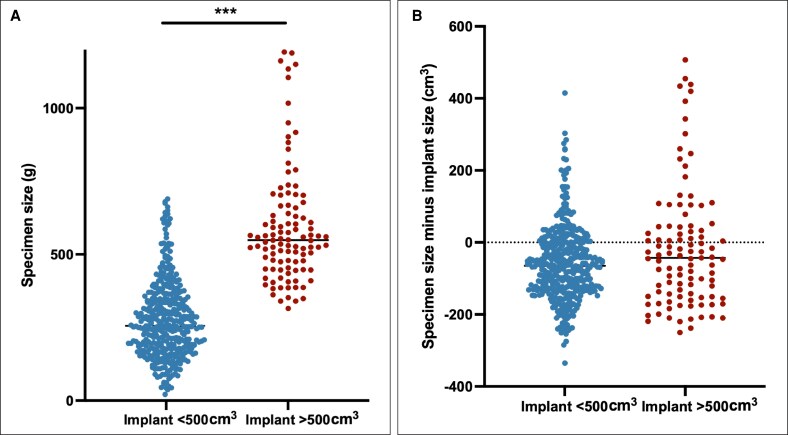
Implant size change by large implant category. (A) Individual specimen size values. ***P < .0001. (B) Individual specimen–implant difference values.

To test the alternative hypothesis that large implant size affects outcomes, data were separated into implant size < 500 cm^3^ or >500 cm^3^. A total of 395 implants were smaller than 500 cm^3^ and 104 were larger than 500 cm^3^. A cutoff point of 500 cm^3^ was used because it was considered “large” for this patient population by the surgeons involved in this study and was ∼1 SD from median implant size in this cohort (median, 375; SD, 140). Implant size > 500 cm^3^ was significantly associated with MSFN (2.28% vs 9.62%, *P* = .0005), seroma (14.4% vs 24%, *P* = .0186), and infection requiring surgery and implant loss (0.51% vs 3.85%, *P* = .0054) by chi-square test (Table 4). The >500 cm^3^ group had a significantly higher proportion of downsize than upsize breasts (26.3% vs 18.0%, *P* = .0116). Specimen size was also significantly higher in the >500 cm^3^ group, as expected since specimen size and implant size are closely correlated (Figure 8A). However, the numerical SID was not significantly different between the <500 cm^3^ and >500 cm^3^ groups (Figure 8B).

**Table 4.** Chi-square Results: <500 cm^3^ Implant vs >500 cm^3^ Implant Groups, Primary and Secondary Outcomes

	Overall %	<500 cm^3^	>500 cm^3^	*P* value
Total		395	104	
MSFN	3.8	9	10	.0005
Seroma	16.4	57	25	.0186
Hematoma	3.0	15	0	.4564
Dehiscence	2.0	6	4	.1319
Infection w/o surgery	3.2	9	7	.0218
Infection w/surgery and implant loss	1.2	2	4	.0054
Downsize	27.7	99	39	.0116

MSFN, mastectomy skin flap necrosis.

Logistic regression was used to model how predictor variables age, BMI, breast cancer, radiation, neoadjuvant chemotherapy, postoperative chemotherapy, and numerical difference between specimen size and implant size affect MSFN rate. Age, breast cancer, radiation, chemotherapy, and numerical specimen-implant size difference were not significantly associated with MSFN. BMI was a significant predictor of MSFN risk (regression coefficient = 0.133997; *P* = .00312).

## DISCUSSION

This single-center, retrospective cohort study of 499 breast reconstructions asked whether the directionality in breast size change influences the risk of MSFN and other complications after immediate postmastectomy DTI reconstruction. We find that downsizing vs upsizing does not significantly affect the risk of MSFN, seroma, or hematoma. Downsizing was associated with significantly increased risk of dehiscence and infection. The downsize group was characterized by significantly higher specimen size, which is known to be associated with increased risk of MSFN.^18,27^

Low rates of mastectomy flap necrosis were observed in this study, with an overall rate of 3.8%. The low rate of necrosis is largely credited to improved techniques by the mastectomy flap surgeons. The 2 mastectomy surgeons receive biannual review and guidance regarding their mastectomy flap necrosis rates and do not perform tumescent technique, which can be associated with increased risk of flap necrosis.^34^ Sharp dissection is performed under the nipple–areola complex to decrease cautery damage in this area of dense and sensitive tissue. Overdissection is rarely or never seen, and there is preservation of perforators from the internal mammary arteries. Where possible, the lateral intercostal perforators are preserved.

Case-by-case examination of the mastectomy flap necrosis patients shows that the majority of patients commonly had necrosis on the lower outer quadrants, just lateral to the skin between the inframammary crease incision and the nipple. Three cases had a thin area of necrotic skin at the incision itself, and 1 patient had full-thickness loss of both nipples. The lower outer quadrant lesions were likely subject to retraction injury, as this area of the mastectomy flap is retracted to allow the mastectomy surgeon to visualize the axillary tail, potentially introducing increased damage to the mastectomy flap.

Our patient population is relatively younger, healthier, and with a lower BMI than the average mastectomy population. Older patients, those with chronic disease, and those with a high BMI may have increased sensitivity to implant size and skin tension and; as with all patients, the individual surgeon's decision-making should prevail.

Our review of cases does not support the hypothesis that a surgically increased breast size is per se associated with higher rates of complication. Rather, absolute implant size is a driver of risk. Despite these suggestive findings, we cannot *affirmatively* claim that increased breast size *does not* increase the risk of MSFN. We cannot rule out a potential false negative result, since post hoc power calculations are dependent on *P* value and should not be used to describe the likelihood of a Type II error.^35,36^ Based on our small observed effect size, a future study of at least 1300 breast reconstructions would be required to confirm (1-b ≥ 0.8) that increasing breast size does not confer additional MSFN risk.

Our results show that the 1 demographic variable that does differ significantly between groups is BMI. BMI was a significant predictor of MSFN in our logistic regression analysis. BMI has previously been identified as an independent predictor of MSFN.^37^ BMI correlates with specimen size, which in turn is significantly different between downsize and upsize groups. Since BMI is a risk factor for MSFN after DTI, 1 alternative explanation for a finding of no difference between downsize and upsize groups is that a generalizable risk from upsizing is being masked by population-specific risk in the downsize group. Although this explanation cannot be disconfirmed in the full dataset, matching to control for BMI in 131 pairs revealed no difference in MSFN rates between the downsizing and upsizing groups, suggesting that higher BMI in the downsize group is not masking MSFN risk due to upsizing.

There is some evidence in this dataset that large implants over 500 cm^3^ confer an independent risk of MSFN and other complications, similar to findings in studies of TE reconstruction.^38^ In support of a null effect of specimen-to-implant size change itself, the numerical difference between specimen and implant does not affect MSFN risk, and the distribution of SID is the same between <500 cm^3^ and >500 cm^3^ groups. It will be important to better characterize the risks of reconstruction with >500 cm^3^ implants, since they are used in a sizable minority of procedures: Approximately 20% of all procedures used >500 cm^3^ implants in this cohort. Moreover, there can be unique advantages to using larger implants in large and ptotic breasts, including reduced rippling and reduced risk of ischemic sequelae arising from loose mastectomy flaps.^39^

The upsize group may have further safety margins to upsize, as this population group, in addition to having a lower BMI, often has breasts that are smaller than a prior size, either from weight loss or prior breastfeeding. This population may tolerate an increase in size just to “fill” the empty space in the breast skin, further decreasing risks in this group. This approach may not only reduce any potential tension on the closure but also decrease the risk of seroma and improve long-term aesthetic outcomes, such as rippling and ptosis.

Since the completion of this study, ICG imaging has been implemented to increase feedback for the mastectomy surgeon and show retraction injury and other areas of low perfusion that may have been preventable. The use of ICG also assists plastic surgeons in assessing flap viability. ICG has recently been used to assess perfusion after initial implant placement in DTI reconstruction, with strong predictive value for MSFN.^31^ Future research should utilize this powerful technique to define the boundaries of safe upsizing.

Overall, we did not find that increasing breast size increases the risk of MSFN. This study is limited by its design, as both single-center and retrospective elements introduce bias. However, since this type of bias generally produces erroneously large effect sizes, our provocative negative result on the risk of downsizing vs upsizing strongly encourages further investigation.^40^ It is unlikely that a larger, multicenter, prospective trial of the contributions of BMI, specimen size, and implant size will alter the recommendation here that upsizing does not incur increased risk of MSFN or other complications. Another important limitation of this study is the lack of validated patient-reported outcome measures, which were not collected and therefore not included in this analysis. Additionally, this cohort was relatively healthy, and cardiovascular fitness measures were not collected. Future research could examine whether the relationship between cardiovascular risk factors and MSFN is altered after upsizing, since underlying microcirculatory vulnerability may increase susceptibility to upsizing-related complications.

Decisions about whether and how to perform DTI are informed by surgeons’ preoperative and intraoperative observations and judgments. Surgical expertise and related intraoperative decisions are the most important drivers of good outcomes in DTI reconstruction.^14^ Complication rates drop precipitously after a surgeon's first year of performing DTI reconstructions.^8^ While numerous studies have investigated patient-centered risk factors, relatively little is known about how reconstructive strategy affects outcomes. For example, intraoperative perfusion mapping decreases MSFN risk and may enable safe placement of larger implants.^41^ Future research, focusing on implant selection and how it interacts with patient characteristics, could enhance evidence-based decision-making in this context. Given the expanding universe of possibility for DTI reconstruction, future work should also investigate how decisions around size change affect the rates of and reasons for revisions.

## CONCLUSIONS

Although current guidelines recommend against increasing breast size during NSM/DTI, we find that increasing breast size did not increase MSFN risk in our single-center retrospective study. Other size-related variables, such as specimen size and implant size, more strongly affect rates of MSFN and other complications. DTI with breast size increase may provide a more aesthetic outcome for many patients and should be considered a viable option for appropriate patients. Flap viability continues to be the most important factor for minimizing complication rates.
